# Machine learning to analyze single‐case graphs: A comparison to visual inspection

**DOI:** 10.1002/jaba.863

**Published:** 2021-07-15

**Authors:** Marc J. Lanovaz, Kieva Hranchuk

**Affiliations:** ^1^ École de psychoéducation Université de Montréal; ^2^ Centre de recherche de l'Institut universitaire en santé mentale de Montréal; ^3^ Behavioural Science and Behavioural Psychology, St. Lawrence College

**Keywords:** AB design, artificial intelligence, machine learning, n‐of‐1 trial, single‐case design, visual analysis

## Abstract

Behavior analysts commonly use visual inspection to analyze single‐case graphs, but studies on its reliability have produced mixed results. To examine this issue, we compared the Type I error rate and power of visual inspection with a novel approach—machine learning. Five expert visual raters analyzed 1,024 simulated AB graphs, which differed on number of points per phase, autocorrelation, trend, variability, and effect size. The ratings were compared to those obtained by the conservative dual‐criteria method and two models derived from machine learning. On average, visual raters agreed with each other on only 75% of graphs. In contrast, both models derived from machine learning showed the best balance between Type I error rate and power while producing more consistent results across different graph characteristics. The results suggest that machine learning may support researchers and practitioners in making fewer errors when analyzing single‐case graphs, but replications remain necessary.

Single‐case designs involve the repeated measurement of an outcome variable within and across different conditions that are defined by the presence or absence of an independent variable (Kratochwill et al., 2010). The central goal of single‐case methodology is to determine whether a causal (i.e., functional) relation exists between the introduction of this independent variable and a change in the dependent variable (Cooper et al., 2020; Horner & Spaulding, 2010). Single‐case research focuses on the individual as the relevant unit of analysis and is appropriate for examining intervention effectiveness (Wolfe et al., 2019). Benefits of single‐case designs over group designs include the study of low‐prevalence disorders (or behavior) that may be difficult to study with traditional group‐design procedures that require a large sample size to yield adequate statistical power (Odom et al., 2005). Furthermore, single‐case designs allow for the analysis of the individual in ways that cannot be facilitated by group designs, where results can be obscured by reporting only group means and their corresponding effect sizes (Horner et al., 2005). From a practical standpoint, single‐case designs are a rigorous option for monitoring and assessing intervention effects in applied settings (Kazdin, 2011).

When using single‐case designs, researchers and practitioners must analyze the resulting graphs to determine whether a functional relation exists between the independent variable and the behavior of interest. Visual inspection remains the most recommended approach for analyzing the results of single‐case designs (Horner et al., 2005; Kazdin, 2011; Ledford & Gast, 2018). This approach involves examining the level, trend, overlap, variability, and immediacy of changes from one condition to another (Kratochwill et al., 2010; Manolov & Vannest, 2020). However, studies on the reliability of visual inspection have produced mixed results (DeProspero & Cohen, 1979; Ford et al., 2020; Kahng et al., 2010; Ninci et al., 2015; Wolfe et al., 2016, 2018). Some researchers have found high agreement between raters (e.g., Ford et al., 2020; Kahng et al., 2010), whereas others have reported concerning results (less than 80% agreement; e.g., DeProspero & Cohen, 1979; Fisher et al., 2003; Wolfe et al., 2016). Some variables that may explain these discrepancies across studies include experience and training of the raters, type of single‐case design displayed within the graph, the presence of contextual details (e.g., target behavior, independent variable, participant information), and whether the raters used a visual aid (Ninci et al., 2015).

Despite some encouraging results (e.g., Ford et al., 2020), one inherent concern with examining interrater agreement alone is that high agreement does not necessarily equate to high validity. High interrater agreement on the analysis of single‐case graphs indicates only that visual raters provided similar ratings on the presence or absence of behavior change. That said, high interrater agreement does not reflect whether these ratings correspond with a *true* effect. In the analysis of single‐case designs, one way to measure validity, or the presence or absence of a true effect, is to examine the accuracy of visual ratings, which can be quantified by Type I error rate and power (Fisher et al., 2003). For visual inspection, accuracy involves the percentage of agreement between the rating of the visual raters (i.e., behavior change vs. no behavior change) and the true effect of the independent variable. Type I error represents a visual rater concluding that a graph shows a clear change when no true behavior change occurred. In contrast, power represents the probability of a visual rater detecting a change in behavior when a true behavior change occurred. For visual inspection to demonstrate strong validity, raters should produce low Type I error rate along with high power, which would in turn be reflected by high accuracy.

To our knowledge, few studies have examined correspondence between visual inspection and true effects (i.e., validity). In a series of studies, Rapp and colleagues (Bartlett et al., 2011; Krueger et al., 2013, Novotny et al., 2014) investigated the validity of visual inspection to analyze single‐case graphs. Their results indicated that visual inspection may produce acceptable Type I error rates (i.e., below .05), but their research did not report power. This omission is concerning because low Type I error rates often result in unacceptably low power (Fisher et al., 2003; Lanovaz et al., 2020). In another notable study, Fisher et al. (2003) found that accuracy of visual raters on simulated AB graphs averaged only 55%. To address this issue, the researchers developed the dual‐criteria method to analyze single‐case graphs. The dual‐criteria method involves tracing a continuation of the mean and trend lines from baseline and examining the number of points falling above (or below) both lines in the treatment phase. The conservative dual‐criteria method is similar, except both lines are moved up (or down) by 0.25 standard deviations, which reduces the Type I error rate. Their visual aid considerably increased the accuracy of visual inspection.

Recently, Lanovaz et al. (2020) compared the accuracy, Type I error rate, and power of four models derived from machine‐learning algorithms to those produced by the dual‐criteria method. Simply put, machine learning involves using sets of instructions to train computers to recognize patterns in data. In the aforementioned study, the researchers trained models to conclude whether an AB graph showed a clear change or not. Their results indicated that all four models outperformed the dual‐criteria method on accuracy, Type I error rate, and power. The main limitation of Lanovaz et al. is that the study did not compare the accuracy, Type I error rate, and power produced by machine learning with those produced by visual inspection. The study only examined correspondence (i.e., agreement) between the two methods, which was high. As more research is conducted, machine learning may become a viable complement, or even alternative, to visual inspection, but a more thorough comparison of the two methods prior to its adoption is necessary. Therefore, the purpose of our study was to address this issue by directly comparing the accuracy, Type I error rate, and power of visual raters and of the conservative dual‐criteria method with those produced by models derived from machine learning on AB graphs with varying characteristics.

## Method

### Dataset

Our dataset contained a total of 1,024 graphs that were simulated using Python 3.7.7. The reader can freely access the data, code, and graphs from our online repository on GitHub: https://github.com/labrl/machine-learning-for-single-case-designs. The file generategraphs.py contains the code that we used to the generate the graphs, whereas GraphsforBlindVisualAnalysis.pdf contains the 1,024 graphs. To examine the effects of different graph characteristics, our code manipulated six different parameters: number of points in Phase A, number of points in Phase B, autocorrelation, trend, variability, and effect size. Table 1 presents the values set for each of those parameters, as well as the number of graphs with each parameter. With the exception of effect size, the values were counterbalanced so that each combination of parameters were equally represented in the dataset. For effect size, we produced fewer graphs with no effect (i.e., an effect size of 0) than graphs with an effect size larger than 0. This manipulation was necessary to test for different effect‐size values. Specifically, the dataset contained 12 graphs for each combination of characteristics showing no effect and 20 graphs for each combination of characteristics showing an effect (i.e., four graphs for each of the five effect sizes larger than 0; see below) for a total 1,024 graphs.

**Table 1:** Characteristics Manipulated Across AB Graphs

Characteristic	Values	
Number of points in Phase A	Shorter: 3 points (*n* = 512) Longer: 5 points (*n* = 512)	
Number of points in Phase B	Shorter: 5 points (*n* = 512) Longer: 10 points (*n* = 512)	
Autocorrelation	Autocorrelation absent: *a* = 0.0 (*n* = 512) Autocorrelation present: *a* = 0.2 (*n* = 512)	
Trend	No trend: 0 degree (*n* = 512) Trend: 30 degrees added (*n* = 512)	
Variability	Stable: variability coefficient = 0.1 (*n* = 512) Variable: variability coefficient = 0.25 (*n* = 512)	
Effect size	No effect: With effect:	SMD = 0 (*n* = 384) SMD = 1 (*n* = 128) SMD = 2 (*n* = 128) SMD = 3 (*n* = 128) SMD = 4 (*n* = 128) SMD = 5 (*n* = 128)

*Note. a*: autocorrelation coefficient, *n*: number of graphs with the value, SMD: standardized mean different.

The number of data points in Phase A was three or five. We chose these values because three points is the minimum number of data points required for a phase in a single‐case design, but guidelines typically recommend five data points (Kratochwill et al., 2010; Ledford & Gast, 2018). For Phase B, the values were set at five and 10 points as (a) practitioners are more likely to have a longer treatment phase than baseline phase, and (b) prior research suggests that having more points in Phase B may reduce Type I error rate and increase power when using the conservative dual‐criteria method (Falligant et al., 2020; Fisher et al., 2003; Lanovaz et al., 2017). For each graph, we began by generating a series of points with a first‐order autocorrelation and an error term randomly generated from a normal distribution with a mean of 0 and a standard deviation of 1. A first‐order autocorrelation represents a correlation between each data point and the data point that immediately precedes it, which is common in single‐case graphs (Shadish & Sullivan, 2011). When present, autocorrelation was set at 0.2, which was the mean first‐order autocorrelation observed in single‐case graphs in a study by Shadish and Sullivan (2011).

To add trend, we used the procedure and values described by Kahng et al. (2010). That is, the function rotated the graph by 30 degrees using the middle point as a pivot. Similarly, the variability coefficients remained the same as those reported by Kahng et al. Given that the standard deviation of the series was 1, we added a constant of 4 to our data series to produce variable graphs (i.e., variability coefficient of 0.25) and a constant of 10 to produce more stable graphs (i.e., variability coefficient of 0.10). It should be noted that changing this parameter is equivalent to manipulating the scale of the y‐axis. Thus, the manipulation may produce a change in visual inspection, but should have no effect on the accuracy of structured aids or machine learning. The final step involved adding a standardized mean difference (SMD) to the points in Phase B to simulate an effect. The value varied from 1 to 5 to represent small to large effect sizes for single‐case graphs (Lanovaz et al., 2019; Levin et al., 2012; Rogers & Graham, 2008).

### Visual Inspection

We recruited five independent raters to conduct the visual inspection component of the study. Each rater held the credential of Board Certified Behavior Analyst‐Doctoral (BCBA‐D), taught in a course sequence verified by the Association for Behavior Analysis International (ABAI), and provided informed consent to participate. Following consent, the authors sent an email consisting of instructions for completing the visual inspection and a .pdf file containing the 1,024 graphs to each rater. The raters inspected each graph individually and responded to the following question, “Would the change observed from Phase A to Phase B be indicative of functional control for an *increase* in behavior if it were reversed and replicated?” The raters recorded a positive response (i.e., 1) when the graph showed a clear change and a negative response (i.e., 0) when the graph showed no clear change. Each AB graph was presented individually on a page and the presentation order of graphs was randomized. The raters had one month to categorize the graphs and enter their values in an Excel file on their computer. To compensate the raters for their time, we offered each rater a $300 online gift card once the task had been completed. Their ratings are available in our repository (see ExpertA.xlsx, ExpertB.xlsx, ExpertC.xlsx, ExpertD.xlsx, and ExpertE.xlsx).

### Conservative Dual‐Criteria

Similarly to Wolfe et al. (2018), our analyses first compared the results of the visual raters with those produced by the conservative dual‐criteria method. For each graph, our code projected a continuation of the baseline mean and trend lines from Phase A onto Phase B. To apply the conservative dual‐criteria method, each line was raised by 0.25 standard deviation. Then, the method involved counting the number of points from Phase B that fell above both lines. Finally, the number of points falling above both lines was compared to two threshold values: 5 when Phase B contained five data points and 8 when Phase B contained 10 data points. If the number of data points was equal to, or exceeded this threshold, the conservative dual‐criteria method considered that graph showed an effect. Otherwise, the graph was categorized as showing no effect.

### Machine Learning Models

Many machine learning algorithms exist to train models to detect effects in single‐case graphs. The current study focused on the two algorithms that showed the most promise according to Lanovaz et al. (2020)—stochastic gradient descent and support vector classifier. Stochastic gradient descent involves applying a function to predict whether a graph shows a change and then fine tuning this prediction by updating weights that multiply the input data during the transformation. As the process repeats itself in a loop, the predictions become more and more accurate in a process similar to shaping in behavior analysis. In the support vector classifier, the algorithm separates the data in higher dimensions (using a hyperplane) to maximize the margin (i.e., separation) between the classes. For more details about these algorithms, we recommend that the reader consult a recent introductory tutorial by Turgeon and Lanovaz (2020) written for behavior analysts.

One issue with the Lanovaz et al. (2020) models is that they were not developed to deal with trend. Therefore, we trained new models using simulated data with similar properties to those being tested. To simulate the data, our code generated 96,000 new graphs using the procedures described in our Dataset section. The only differences were that (a) more graphs were generated to improve the accuracy of the models, and (b) an equal number of graphs with and without effects were produced. That is, half the graphs had an SMD of 0, whereas the other half had an SMD varying from 1 to 5 (equally distributed). Length of Phase A (three or five points), length of Phase B (five or 10 points), autocorrelation (0 or 0.2), trend (0 or 30 degrees) and variability coefficient (.10 or .25) remained perfectly counterbalanced. Having 96,000 graphs allowed us to have at least 300 exemplars for each combination of characteristics, which was necessary as machine learning requires large datasets to produce accurate predictions. The file “trainingmodels.py” in our repository contains all the code necessary to replicate the procedures used to train our model.

Despite sharing a similar distribution, the graphs used for training the machine‐learning models (i.e., the 96,000 graphs) were not the same as those analyzed by the behavior analysts (i.e., the 1,024 graphs) so that we could test the trained models for generalization on the untrained graphs analyzed by the behavior analysts. The 96,000 graphs were used to train the models using the same code and procedures as described by Lanovaz et al. (2020). The only change was that our code used a hinge‐loss function rather than a logistic‐loss function for the stochastic gradient descent because research suggests that the former may produce more accurate results (Barbu et al., 2018; Rosasco et al., 2004). First, a function standardized the data so that each graph had a mean of 0 and a standard deviation of 1. Second, the code extracted eight features from each graph: mean of Phase A, mean of Phase B, standard deviation of Phase A, standard deviation of Phase B, intercept of Phase A, slope of Phase A, intercept of Phase B, and slope of Phase B. The class labels represented no change (i.e., no SMD added) and change (SMD added). Finally, the algorithms used these features and the class labels to train the models (see trainingmodels.py for our code).

### Analyses

The first step involved applying the conservative dual‐criteria method and the machine‐learning models to the 1,024 graphs analyzed by the behavior analysts. This analysis produced a series of vectors where 0 represented no change detected by the analysis and 1 represented a detected change (see cdc_values.csv, sgd_values.csv, and svc_values.csv). Next, our procedures examined accuracy as well as pairwise agreement between all raters and methods of analyses (see comparisons.py). Accuracy represented the percentage of agreement (i.e., number of agreements divided by total number of graphs analyzed) between the true values and each method of analysis. The true values indicated whether we added a simulated effect to the graph; the value was set at 0 (i.e., no effect) when no SMD had been added to the graph and at 1 (i.e., effect) when an SMD value of 1 to 5 had been added to Phase B of the graph (see true_values.csv). Then, the analyses involved examining Type I error rate and power. Our function computed Type I error rate by dividing the number of times that the method of analysis indicated an effect in graphs that show no true effect by the total number of graphs showing no true effect. For power, our code instructed Python to add the number of times that the analyses detected an effect in graphs showing a true effect and to divide this sum by the total number of graphs showing a true effect. Finally, we repeated the analyses to examine the effects of manipulating six graph characteristics: number of points in Phase A, number of points in phase B, autocorrelation, trend, variability, and effect size. These analyses also involved the computation of the 95% confidence interval for the binomial distribution. If a mean fell outside the confidence interval of its comparison value, we considered the difference between the two to be significant.

## Results

**Figure 1:**
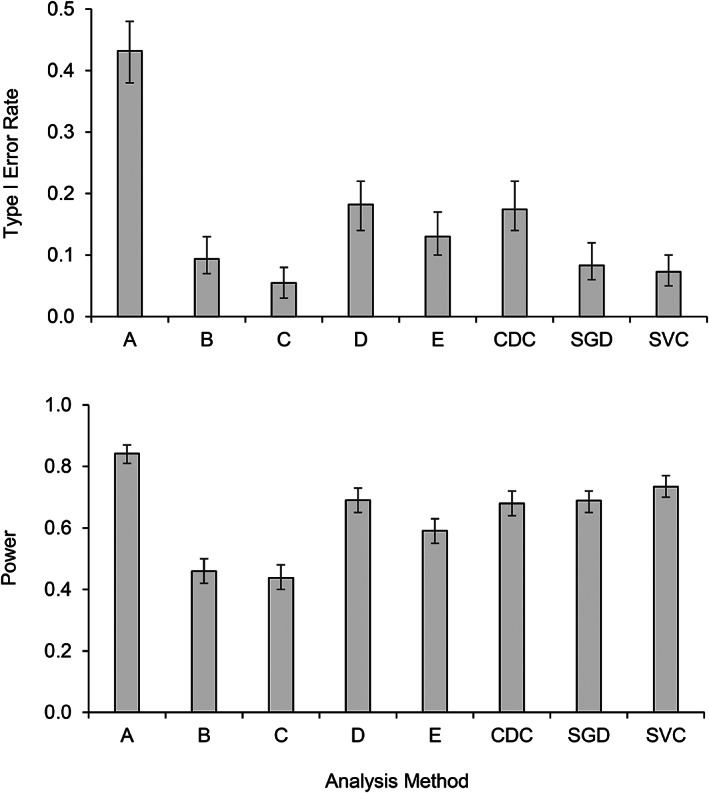
Type I Error Rate and Power for Each Method of Analysis *Note*. CDC: conservative dual‐criteria, SGD: stochastic gradient descent, SVC: support vector classifier. The vertical bars represent 95% confidence intervals.

Table 2 presents agreement between the true values and the different methods of analyses. The first column represents the accuracy of each method. The two methods of analyses with the highest accuracy were those derived from machine learning—stochastic gradient descent (.77) and support vector classifier (.81). The accuracy of the visual raters ranged from .63 to .74. Agreement between raters varied between .59 and .86 with a mean of .75. Figure 1 presents a fine‐grained analysis of the errors produced by each method. The upper panel of Figure 1 shows that Expert B, Expert C, the stochastic gradient descent, and the support vector machine produced the lowest Type I error rates (see upper panel of Figure 1). On the other hand, Expert A produced the highest power, whereas Expert D, the conservative dual‐criteria method, the stochastic gradient descent, and the support vector classifier were closely matched in second place (see lower panel of Figure 1). Concerningly, the expert with the lowest Type I error rate (Expert C) also had the lowest power. Similarly, the expert with highest power (Expert A) also had the highest Type I error rate. These patterns underline a challenge faced by both visual raters and structured methods of analysis: When an analysis is made more stringent to reduce Type I error rates (i.e., false positives), this manipulation tends to produce an increase in Type II error rates (i.e., false negatives), which reduces power. The methods that offered the best balance between Type I error rate and power are also the most accurate ones—the stochastic gradient descent and the support vector classifier.

**Table 2:** Proportion of Correspondence Between the True Values and Different Methods of Analyses

	True	Expert A	Expert B	Expert C	Expert D	Expert E	CDC Method	SGD
Expert A	.74							
Expert B	.63	.61						
Expert C	.63	.59	.86					
Expert D	.74	.79	.79	.78				
Expert E	.70	.69	.79	.80	.82			
CDC Method	.73	.73	.75	.75	.80	.90		
SGD	.77	.73	.76	.77	.83	.78	.76	
SVC	.81	.72	.75	.75	.82	.79	.76	.93

*Note*. CDC: conservative dual‐criteria, SGD: stochastic gradient descent, SVC: support vector classifier.

**Figure 2:**
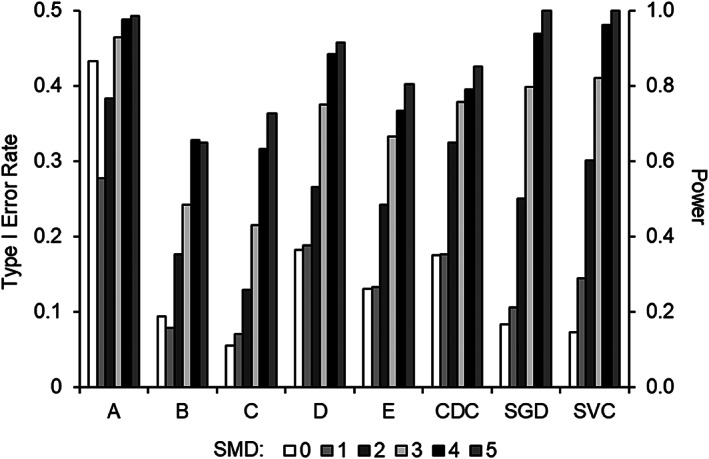
Type I Error Rate (SMD = 0) and Power (SMD = 1 to 5) Across Standardized Mean Differences for Each Method of Analysis *Note*. CDC: conservative dual‐criteria, SGD: stochastic gradient descent, SVC: support vector classifier, SMD: standardized mean difference.

Figure 2 depicts Type I error rate and power across different SMD values. Excluding Expert A (who had unacceptably high Type I error rates), the two methods that produced the highest power with an effects size of 3 or more were the stochastic gradient descent and the support vector classifier. This observation is important because most single‐case designs showing functional relations have SMD values higher than 3 (Lanovaz et al., 2019; Levin et al., 2012; Rogers & Graham, 2008).

The Supporting Information for this article contains a series of tables that examine the effects of each graph characteristic on Type I error rate and power across methods of analysis. The next paragraphs summarize the main findings contained in these tables. For the number of points in Phase A, only Experts B and C showed significantly less Type I error when Phase A contained more points. The effects on power were mixed; Expert E, the conservative dual‐criteria method, and the support vector classifier produced more power when Phase A had more points, whereas the stochastic gradient descent displayed the opposite pattern. For the length of Phase B, three raters (i.e., Experts A, C, and E) and the conservative dual‐criteria method unexpectedly produced more Type I error on graphs that had 10 points in Phase B than on those that had only five points. Interestingly, this increase in Type I error did not result in more power with only Expert D showing higher power on shorter graphs. Furthermore, our results indicate that autocorrelation had no significant effect on Type I error rate. Only Expert B displayed a significant difference with power; the participant had more power when no autocorrelation was present.

Contrarily, adding trend increased the Type I error for all participants and methods of analyses but to a lesser extent for the two machine learning algorithms, as well as for Experts B and C. With the exception of the support vector machine, all approaches showed more power in the presence than in the absence of trend. This result is concerning because it means that the overall power values presented for the visual raters in Figure 1 were inflated by the presence of trend. As expected, altering variability had no effect on Type I error rate and power for the structured methods (i.e., conservative dual‐criteria method, stochastic gradient descent, and support vector classifier). In contrast, three of five raters produced significantly more Type I error when the graphs showed more variability, and all raters had more power for more variable data. Again, this result raises serious concerns about the power of visual inspection when variability is low.

## Discussion

Overall, our results indicate that models derived from machine learning generally produced fewer errors than visual raters and the conservative dual‐criteria method. Moreover, the support vector classifier and stochastic gradient descent were generally less affected by changing characteristics in the data than were visual raters. The support vector classifier appears to produce marginally lower Type I error rate and more power than the stochastic gradient descent. These results are consistent with Lanovaz et al. (2020), who showed that machine learning produces less error than the dual‐criteria method. The current study extends this finding by showing that machine learning may also perform better than visual raters. By far, the most consequential characteristic manipulated was trend. All methods of analyses produced more Type I errors on datasets with trend. Apart from the support vector classifier, most methods also produced less power in the absence of trend.

Our study also contributes to work on the reliability and validity of visual inspection for single‐case graphs. First, agreement between raters was moderate at best, even though all participants had similarly advanced credentials (i.e., BCBA‐D certification) and taught in programs designed to train future behavior analysts. This observation raises concerns regarding the reliability and validity of visual inspection. Visual raters seem to struggle with balancing Type I error with power. This issue raises questions regarding results reported by Rapp and colleagues (Bartlett et al., 2011; Krueger et al., 2013, Novotny et al., 2014), who did not evaluate power. This fine balance between Type I error rate and power is well illustrated in patterns that we observed with trend and variability. Having no trend decreased Type I error rate, but it also reduced power. Along the same line, having less variability also decreased both Type I error rate and power.

The method used to simulate graphs and effects may explain some of the errors produced by the visual raters. To simulate an effect, we added an SMD value that varied between 1 and 5. In contrast, the value of data points with no autocorrelation fell within plus or minus 1 of the mean in 68% of cases and within plus or minus 2 of the mean in 95% of cases. A 30‐degree trend also added or subtracted 0.58 to each point, sequentially. Therefore, random variation alone could mask the programmed trend and small effect sizes, especially when both phases contained the minimum number of points. The visual raters had to see beyond this “noise” in the data to identify effects accurately, which could have been a challenge. Nonetheless, the machine‐learning algorithms had more power than four of the five raters even with high effect sizes (a hallmark of applied behavior analysis). The only rater who outperformed the algorithms on power (i.e., Expert A) also had unacceptably high Type I error rates (i.e., in excess of 40%).

The lower validity and moderate agreement of the expert raters raise a controversial question for behavior analysts—should we trust visual inspection at its face value? The problem is that each visual rater produces different patterns of errors. Therefore, researchers and practitioners remain unaware of their own error rate and power unless they participate in a study like the one described here. One potential solution is to train behavior analysts to conduct visual inspection using simulated graphs with known class labels (i.e., change or no change). Training could terminate when raters show acceptable levels of errors based on untrained exemplars (as in multiple‐exemplar training). An alternative is to use systematic protocols or procedures (e.g., Manolov & Vannest, 2020; Wolfe et al., 2019) and examine whether they improve the validity of the analyses of raters using them. Given that behavior analysts put much emphasis on interobserver agreement, calibrating visual inspection appears to be a logical next step for improving the validity of visual inspection.

The current results and those reported by Lanovaz et al. (2020) support the use of machine learning to develop models to complement the visual inspection of single‐case graphs. Using machine learning may support raters in reducing their Type I error rate while increasing power. One potential barrier to the adoption of machine learning in behavior analysis is the lack of training in the area. The techniques used to the develop the models are likely too quantitatively advanced to be used by clinicians without explicit training in machine learning, computer science, and/or statistics. To address this issue, researchers may develop online calculators that simply take the data points and conditions as input and produce an output with a probability (see https://labrl.shinyapps.io/singlecaseanalysis/ for example from Lanovaz et al., 2020) to facilitate the use of these algorithms.

Another concern involves the use of AB designs in research. That is, AB designs do not allow for the demonstration of functional control. As recommended by Fisher et al. (2003) for the dual‐criteria methods, researchers could apply the current machine‐learning models to phase changes in reversal and multiple‐baseline graphs. Moreover, some practitioners may rely on AB designs due to practical and ethical considerations (Lanovaz et al., 2019). A related limitation to this issue involves the wording of the question asked to the reviewers, “Would the change observed from Phase A to Phase B be indicative of functional control for an increase in behavior if it were reversed and replicated?” The latter was necessary because AB graphs cannot show functional control. That said, this manipulation requires that the raters imagine the rest of the graph on their own, which may have added an unintended confound to our analyses.

A question raised by the current study is the extent to which machine learning could handle ongoing data analysis. Our procedures did not consider patterns when simulating sessions. The phase lengths were preset regardless of observed trends, which is inconsistent with the response‐guided, decision‐making process often used by behavior analysts. The models developed as part of the current study could be applied repeatedly as treatment points are added to the design, but this approach may inflate Type I error rate (as with any repeated application of the conservative dual‐criteria method). To address this issue, further simulation studies could use an objective response‐guided method (e.g., fail safe *k* metric; Barnard‐Brak et al., 2018) to simulate graphs. Alternatively, researchers could embed this decision‐making process within the machine‐learning algorithm itself.

Our study has other limitations that should be noted. First, the study focused on simulated data rather than nonsimulated data. Simulated graphs may not perfectly capture patterns observed in research and clinical data, whereas nonsimulated graphs do not allow for the examination of power. In the future, one solution to this problem could involve asking the expert raters to score the realism of the simulated graphs (akin to the assessment of social validity for interventions). For example, researchers could ask the following question to raters, “On a scale of 1 to 5 (1 = totally agree, 5 = totally disagree), to what extent do you agree with the following statement: The patterns observed in the current graph resemble those that I have encountered in practice or research.” An alternative could involve setting a discrimination task and asking raters to identify simulated graphs amongst an array of nonsimulated and simulated graphs. A correct response rate close to chance would indicate that the simulated graphs closely matched real patterns of behavior. Then, only simulated graphs that closely mimic patterns already observed by the raters would be kept for subsequent analyses.

Finally, the applicability of our models is restricted by the distribution of the data used for training and testing. Researchers have two options to address this limitation: (a) test the models that we developed in the current study on novel datasets or (b) develop and test new models. The first option is easier, as the researchers would not have to train novel models. For example, researchers could extract 300 graphs from theses and dissertations, and examine the extent to which the current models produce results consistent with visual raters. We would advise against using graphs published in peer‐reviewed journals because such datasets may result in an insufficient number of graphs showing no effect (Dowdy et al., 2020; Sham & Smith, 2014). Having a large number of graphs is typically better because it reduces the size of the confidence intervals of the results. As an example, the 95% confidence interval produced by the visual rating of 300 graphs is approximately plus or minus .05 for a power of .80. To prevent the proliferation of models, our recommendation is to test existing models first. If existing models produce poor validity, a second option involves training new models, which is more complex and requires additional data (see Turgeon & Lanovaz, 2020, for a tutorial). Lanovaz et al. (2020) used more than 1,000 graphs to train their models. The problem is that the dataset must be separated in two; some graphs train the models, whereas the remaining graphs test for generalization. Regardless of the approach adopted, researchers must replicate our study with graphs with other distributions and characteristics to ensure that the results remain consistent and generalizable prior to the adoption of machine learning to complement the analysis of single‐case graphs.

## Supporting information


**Appendix S1**: Supporting information
