# Supplementary material for: Machine learning to analyze single‐case graphs: A comparison to visual inspection
Source: J Appl Behav Anal. 2021 Jul 15;54(4):1541–52. doi: 10.1002/jaba.863 (PMC8596748; doi:10.1002/jaba.863)
Supplement: Supplementary file 1 — Appendix S1: Supporting information [file JABA-54-1541-s001.docx]

**Supporting Information**

**Table A1**

*Type I Error Rate and Power for Each Method Across Different Lengths of Phase A*

|  | Type I Error Rate | |  | Power | |
| --- | --- | --- | --- | --- | --- |
|  | 3 data points | 5 data points |  | 3 data points | 5 data points |
|  |  |  |  |  |  |
| Expert A | .46 [.39, .53] | .41 [.34, .48] |  | .83 [.79, .87] | .85 [.81, .89] |
| Expert B | .12 [.08, .17] | .07 [.04, .11] |  | .48 [.42, .53] | .44 [.39, .50] |
| Expert C | .08 [.04, .13] | .03 [.01, .07] |  | .46 [.40, .52] | .42 [.36, .47] |
| Expert D | .20 [.15, .27] | .16 [.11, .22] |  | .70 [.64, .75] | .68 [.63, .73] |
| Expert E | .11 [.07, .17] | .15 [.10, .20] |  | .56 [.50., .61] | .62 [.57, .68] |
| CDC Method | .16 [.11, .22] | .19 [.13, .25] |  | .63 [.58, .68] | .73 [.68, .78] |
| SGD | .09 [.05, .14] | .08 [.04, .13] |  | .72 [.66, .76] | .66 [.61, .71] |
| SVC | .07 [.04, .12] | .07 [.04, .12] |  | .70 [.64, .75] | .77 [.72, .82] |

*Note.* CDC: conservative dual-criteria, SGD: stochastic gradient descent, SVC: support vector classifier**Table A2**

*Type I Error Rate and Power for Each Method Across Different Lengths of Phase B*

|  | Type I Error Rate | |  | Power | |
| --- | --- | --- | --- | --- | --- |
|  | 5 data points | 10 data points |  | 5 data points | 10 data points |
|  |  |  |  |  |  |
| Expert A | .37 [.30, .44] | .49 [.42, .57] |  | .85 [.81, .86] | .83 [.79, .87] |
| Expert B | .08 [.05, .13] | .10 [.06, .16] |  | .48 [.42, .53] | .44 [.39, .50] |
| Expert C | .03 [.01, .07] | .08 [.04, .13] |  | .44 [.39, .50] | .43 [.38, .46] |
| Expert D | .18 [.13, .24] | .18 [.13, .24] |  | .73 [.68, .78] | .65 [.60, .71] |
| Expert E | .09 [.06, .14] | .17 [.12, .23] |  | .59 [.53, .65] | .59 [.53, .65] |
| CDC Method | .12 [.08, .17] | .23 [.17, .30] |  | .68 [.63, .73] | .68 [.62, .73] |
| SGD | .10 [.06, .16] | .06 [.03, .11] |  | .68 [.63, .73] | .69 [.64, .74] |
| SVC | .08 [.05, .13] | .06 [.03, .11] |  | .74 [.69, .78] | .73 [.68, .78] |

*Note.* CDC: conservative dual-criteria, SGD: stochastic gradient descent, SVC: support vector classifier

**Table A3**

*Type I Error Rate and Power for Each Method in the Presence and Absence of Autocorrelation*

|  | Type I Error Rate | |  | Power | |
| --- | --- | --- | --- | --- | --- |
|  | *a* = 0 | *a* = 0.2 |  | *a* = 0 | *a* = 0.2 |
|  |  |  |  |  |  |
| Expert A | .43 [.36, .51] | .43 [.36, .51] |  | .84 [.80, .88] | .84 [.80, .88] |
| Expert B | .10 [.06, .16] | .08 [.05, .13] |  | .50 [.44,.56] | .42 [.36, .47] |
| Expert C | .06 [.03, .11] | .05 [.02, .08] |  | .47 [.41, .52] | .41 [.35, .47] |
| Expert D | .19 [.13, .25] | .18 [.13, .24] |  | .70 [.65, .75] | .68 [.63, .73] |
| Expert E | .14 [.09, .19] | .13 [.08, .18] |  | .60 [.54., .65] | .58 [.53, .64] |
| CDC Method | .17 [.12, .23] | .18 [.13, .24] |  | .67 [.61, .72] | .69 [.64, .74] |
| SGD | .09 [.05, .14] | .08 [.04, .13] |  | .69 [.64, .74] | .69 [.63, .74] |
| SVC | .07 [.04, .12] | .07 [.04, .12] |  | .73 [.68, .78] | .73 [.68, .78] |

*Note.* CDC: conservative dual-criteria, SGD: stochastic gradient descent, SVC: support vector classifier, *a*: autocorrelation

**Table A4**

*Type I Error Rate and Power for Each Method in the Presence and Absence of Trend*

|  | Type I Error Rate | |  | Power | |
| --- | --- | --- | --- | --- | --- |
|  | *t* = 0 | *t* = 30 |  | *t* = 0 | *t* = 30 |
|  |  |  |  |  |  |
| Expert A | .07 [.04, .12] | .79 [.73, .85] |  | .71 [.66, .76] | .97 [.95, .99] |
| Expert B | .02 [.01, .05] | .17 [.12, .23] |  | .42 [.36,.47] | .50 [.44, .56] |
| Expert C | .00 [.00, .02] | .11 [.07, .16] |  | .38 [.32, .43] | .50 [.44, .56] |
| Expert D | .01 [.01, .04] | .35 [.29, .43] |  | .61 [.55, .66] | .78 [.73, .82] |
| Expert E | .03 [.01, .06] | .23 [.18, .30] |  | .52 [.46., .57] | .66 [.61, .71] |
| CDC Method | .05 [.03, .09] | .30 [.23, .37] |  | .61 [.55, .66] | .75 [.70, .80] |
| SGD | .01 [.01, .04] | .16 [.11, .22] |  | .63 [.58, .69] | .74 [.69, .79] |
| SVC | .02 [.01, .04] | .13 [.09, .19] |  | .75 [.70, .80] | .72 [.67, .77] |

*Note.* CDC: conservative dual-criteria, SGD: stochastic gradient descent, SVC: support vector classifier, *t*: trend

**Table A5**

*Type I Error Rate and Power for Each Method Across Different Levels of Variability*

|  | Type I Error Rate | |  | Power | |
| --- | --- | --- | --- | --- | --- |
|  | *v* = .10 | *v* = .25 |  | *v* = .10 | *v* = .25 |
|  |  |  |  |  |  |
| Expert A | .41 [.34, .48] | .46 [.39, .53] |  | .79 [.75, .84] | .89 [.85, .92] |
| Expert B | .06 [.03, .10] | .13 [.09, .19] |  | .35 [.30, .40] | .57 [.51, .62] |
| Expert C | .02 [.01, .04] | .09 [.06, .14] |  | .33 [.28, .38] | .55 [.49, .60] |
| Expert D | .14 [.09, .20] | .22 [.17, .29] |  | .58 [.53, .64] | .80 [.75, .84] |
| Expert E | .11 [.07, .17] | .15 [.10, .20] |  | .55 [.49, .61] | .63 [.58., .68] |
| CDC Method | .19 [.14, .26] | .16 [.11, .22] |  | .66 [.61, .71] | .70 [.64, .75] |
| SGD | .07 [.04, .12] | .09 [.06, .14] |  | .67 [.62, .72] | .71 [.65, .76] |
| SVC | .07 [.04, .11] | .08 [.04, .13] |  | .73 [.67, .77] | .74 [.69, .79] |

*Note.* CDC: conservative dual-criteria, SGD: stochastic gradient descent, SVC: support vector classifier, *v*: variability coefficient
